# Specialist Palliative Care Consultations in COVID-19 Patients in the ICU—A Retrospective Analysis of Patient Characteristics and Symptoms at a German University Hospital

**DOI:** 10.3390/jcm11195925

**Published:** 2022-10-07

**Authors:** Theresa Tenge, Sebastian Brimah, Daniel Schlieper, Antje Roesel, Jacqueline Schwartz, Manuela Schallenburger, Stefan Meier, Timo Brandenburger, Detlef Kindgen-Milles, Peter Kienbaum, Martin Neukirchen

**Affiliations:** 1 Department of Anesthesiology, Medical Faculty and University Hospital Duesseldorf, Heinrich-Heine-University Duesseldorf, 40225 Duesseldorf, Germany; 2 Interdisciplinary Center for Palliative Medicine, Medical Faculty and University Hospital Duesseldorf, Heinrich-Heine-University Duesseldorf, 40225 Duesseldorf, Germany

**Keywords:** palliative care, patient-centered care, intensive care unit, pandemic, COVID-19, interprofessional care, symptom control, interdisciplinary care

## Abstract

COVID-19 patients who may require invasive therapeutic procedures such as extracorporeal membrane oxygenation (ECMO) have high symptom burden and in-hospital mortality. In addition, awake patients on ECMO are new in the intensive care unit (ICU) setting. Inpatient specialist palliative care (sPC) provides support such as symptom control on a physical, psychosocial and spiritual level. The field of sPC in COVID-19 patients is still new and important to investigate. We aim to analyze sPC of COVID-19 patients in the ICU with regard to patient characteristics and symptoms from a palliative care perspective. We conducted a retrospective analysis (03/2020–04/2021) and identified 51 ICU patients receiving sPC. The statistical analysis included descriptive statistics and comparisons of symptoms. The first sPC contact of patients (mean age 69.5 years, 62.7% male) was around 14 days after COVID-19 confirmation, and 43% were treated with ECMO therapy. The baseline symptom burden was high with a focus on weakness (100%), tiredness (98%), dyspnea (96%) and family burden (92%). The symptom intensity significantly decreased during the time period of sPC and COVID-19 treatment (t(99) = 3.119, *p* = 0.003, d = 0.437). These results help intensivists and sPC clinicians to identify symptoms and the need for sPC in COVID-19 patients. However, studies with prospective and controlled designs need to follow.

## 1. Introduction

Since the first reports at the end of 2019, the severe acute respiratory syndrome coronavirus type 2 (SARS-CoV-2) causing the coronavirus disease 2019 (COVID-19) has spread around the world and was classified as a pandemic by the World Health Organization (WHO, Geneva, Switzerland) in March 2020 [1]. Since then, the COVID-19 pandemic has affected the human population, with a massive impact on all aspects of everyday life including healthcare systems and research. A rapidly increasing number of patients suffered from the new disease; many died, and relatives suddenly lost their beloved ones. Meanwhile, our knowledge about the symptoms and mortality was developing. The current community-based data from a longitudinal prospective study regarding the periods of the dominance of the Omicron and the Delta variants describe the most frequent symptoms as a runny nose, headache, sneezing, sore throat and loss of smell [2]. The case fatality is estimated at 0.1% in Australia, 0.5% in Germany, 0.8% in the United Kingdom, 1.1% in the United States and 2.5% in South Africa [3]. To date, more than 140,000 patients have died in Germany, and in the United States, more than one million people have lost their lives [3]. For patients admitted to intensive care units (ICUs), the estimated mortality is up to 30% with higher rates in older patients [4,5]. For patients receiving extracorporeal membrane oxygenation (ECMO), mortality is even higher with a range between 37% and 73% depending on the centers and countries [6,7,8]. The recent data described COVID-19 ECMO mortality to be higher in Germany than elsewhere in Western countries; the possible reasons might be due to the availability and more frequent usage of ECMO therapy in older patients compared to other countries [7,8].

Since the beginning of the COVID-19 pandemic, clinicians and researchers have explored the use and roles of general and specialist palliative care (PC) in COVID-19 patients [9]. While all medical professions and disciplines provide general PC, specialist PC (sPC) is provided by a multiprofessional sPC team. Briefly, PC not only aims to support patients with life-limiting diseases but also their relatives. With the assessment of all, physical, psychosocial and spiritual domains, the improvement of quality of life is the main focus. In general, studies have shown numerous positive effects of (s)PC, including improved symptom control, better quality of life and even prolonged survival in cancer patients [10,11,12]. For hospitalized patients, in addition to admission to sPC units, patients may receive regular palliative care complex care visits from the multiprofessional sPC teams on normal or intensive care units. Right at the beginning of the pandemic, the constant demand for inpatient PC was assessed, and the sPC teams responded to the pandemic situation, e.g., using digital telehealthcare approaches [13,14,15,16,17]. However, the general PC and sPC infrastructure also suffered, e.g., through staff shortages, strict visiting regulations, impaired communication within the team and with patients and their families [18,19,20,21,22,23,24]. In addition, some sPC units and hospices were closed [13,21]. The review articles summarize the rapid developing publications and statements on PC need and challenges in treating COVID-19 patients [25,26,27,28]. Moreover, sPC support within the ICU teams was addressed [29]. sPC involvement in COVID-19 patients was first described in a case series by Lovell and colleagues [30]. The 101 COVID-19 patients with referral to sPC in their cohort were mostly male with a mean age of 82 years, and 67% of this cohort suffered from breathlessness [30]. After a median of three sPC contacts, 74% of the patients died during the short study period of one month [30]. Another retrospective analysis of hospitalized COVID-19 patients showed sPC involvement in 17% of all and 48% of the deceased patients [31]. Golob and colleagues investigated the prevalence and association of sPC with end-of-life care in deceased COVID-19 patients from February until April 2020 [32]. Of the total 203 patients (61% male, median age 76 years), 56% received sPC and were thereby less likely to undergo invasive procedures or life-sustaining treatments and spent less time in the ICU at the end of life [32]. However, other studies revealed higher rates of life-sustaining treatments in patients with sPC [33,34]. A multicenter study on critically ill COVID-19 patients revealed no influence of sPC consultations on the initiation of cardiopulmonary resuscitation rates but found more comfort care with better symptom control and individual social, psychological and spiritual support at end-of-life [35]. Finding predictors of inpatient sPC consultations in COVID-19 patients was investigated by Frydman et al. [36]. The retrospective analysis showed an association of age, serious illness such as cancer, chronic obstructive pulmonary disease, dementia and greater illness severity to sPC consultations [36]. In patients aged 50 years or older, Almeida and colleagues found age ≥ 80 years, dementia, history of stroke or cancer, frailty, PaO_2_/FiO_2_ < 200 or C-reactive protein ≥ 150 mg/dL at admission were predictors of sPC implementation [37]. The overall sPC involvement rate in their retrospective cohort from March to May 2020 was 17% [37].

As COVID-19 is still a new and emerging disease, we need to further explore the use and demand of sPC in hospitalized patients in ICU with high mortality rates. The aim of this study is to describe and investigate the involvement of sPC in COVID-19 patients in ICU during the pandemic with regard to patient characteristics and symptoms from a palliative care perspective. These characteristics can help clinicians in the future to identify COVID-19 patients who may benefit from sPC involvement. In particular, the analysis of the specific symptom burden and symptom intensities of these patients may provide guidance for clinicians.

## 2. Materials and Methods

### 2.1. Study Design and Ethical Approval

We conducted a retrospective, single-center study at the university hospital in Duesseldorf, Germany to investigate inpatient sPC involvement in COVID-19 patients in the ICU. The study was approved by the local ethics committee of the Medical Faculty of Heinrich-Heine-University Duesseldorf, Germany (study no. 2021-1501). Writing of this article was performed following the STROBE guidelines [38].

### 2.2. COVID-19 Setting and Specialist Palliative Care

At the university hospital Duesseldorf with more than 50,000 inpatient cases per year, the first COVID-19 patients were treated in February 2020. Until the end of our study period in April 2021, 985 patients with COVID-19 have been treated on standard and intensive care units. The hospital has a long-established sPC service with an eight-bed sPC unit, an inpatient sPC consultation team and an outpatient sPC team. Inpatients sPC involvement consists of patient contact with sPC nurses, sPC physicians, and, if needed, with other sPC team members (e.g., psychological and social support, physiotherapy and massage, creative therapy, spiritual care and support by volunteers). The team has regular multiprofessional discussions about the patient needs and treatment goals. sPC consultation is initiated on request of the primary caregivers via the electronic hospital information system used in our institution (CGM MEDICO^®^, Koblenz, Germany). The baseline sPC assessment is performed within 24 h. The following sPC treatment is adapted in frequency, intensity and organization to the needs of patients and their families with regard to the physical, psychosocial and spiritual problems. After the first experiences of treating COVID-19 ICU patients, a consensus on the need of sPC in COVID-19 patients with tracheostomy due to a long time need for mechanical ventilation and ECMO treatment was reached. However, no thresholds for sPC consultation in these patients was provided.

### 2.3. Patient Selection and Data Collection

Data were obtained from the electronic patient data system. Inclusion criteria were all adult COVID-19 patients as confirmed by positive polymerase chain reaction tests, treated in the ICU receiving inpatient sPC from March 2020 until April 2021. In this time period, the first COVID-19 wave occurred from March until May 2020, followed by a period with low case numbers during the summer. The second wave took place from the end of September 2020 until February 2021, with a third wave immediately following which lasted until June 2021 [39]. Exclusion criteria were age younger than 18 years and incomplete medical records with missing data regarding the relevant information. Data collection included the following information which is routinely documented by the sPC teams during their treatment and was retrospectively assessed from the medical records by SB: age, gender, place of living, Eastern Cooperative Oncology Group (ECOG) status (originally developed for cancer patients; six grades; from 0 = unrestricted activity to 5 = death), availability of a close connection to relatives and the presence of advance directives or a designated healthcare proxy and comorbidities. Furthermore, we determined the date of the first SARS-CoV-19 confirmation test, the date of first sPC contact and the different interventions used to treat COVID-19 during sPC involvement. If a patient died during the course of the sPC treatment, the date of death was identified. In addition, we recorded the presence and intensity of symptoms using the German minimal documentation system (MIDOS) assessment score. [40,41]. This score is routinely used for every sPC treatment baseline and discharge assessment in our hospital and documented by the sPC nurses in the medical record. In line with a previous study [42], symptom intensity (minimum: 0, maximum: 48) was measured by summing up the severity (0 = no symptoms, 1 = light, 2 = moderate, 3 = severe) of each of the 16 items [42].

### 2.4. Data Analysis

Descriptive and further statistical analyses were performed using IBM SPSS Statistics (version 28.0, IBM Corp, Armonk, NY, USA). Shapiro–Wilk test was used to test for normality in symptom intensity at the beginning and end of sPC. The comparison of symptom intensities was obtained using a 2-sample paired *t*-test. A two-sided *p*-value of <0.05 was considered to indicate statistical significance. Continuous variables are presented by the mean and standard deviation (SD), whereas categorical variables are shown as absolute and relative (%) frequencies. The figures were generated using Microsoft Excel (version 16.42, Microsoft Corp., Redmond, WA, USA).

## 3. Results

### 3.1. Patient Characteristics

A total of 51 COVID-19 patients in the ICU received sPC from March 2020 until April 2021. During this period, 262 COVID-19 patients were in the ICU in general, 53 of the latter were receiving ECMO therapy (20.2%). The mean duration of ECMO therapy in COVID-19 patients during this time was 12.1 days, with a range from one to 46 days. In this study, 19.5% (51/262) of all ICU COVID-19 patients and 43.4% (23/53) of all ECMO patients received sPC. The mean age of the 51 included patients was 69.5 years, and 62% were male. Most patients (82.4%) lived at home, and 94.1% had regular contact with their relatives before hospital admission. The most common comorbidity was arterial hypertension (70.6%), followed by coronary artery disease (39.2%) and obesity (31.4%). One in four patients suffered from an oncological disease (25.5%). Advance directives were present in one quarter of all patients (25.5%) and healthcare proxies in almost half of all patients (45.1%). The patient characteristics with information unrelated to COVID-19 are given in Table 1.

### 3.2. Specialist Palliative Care Involvement

At the time of first sPC contact, patients had, on average, tested positive for SARS-CoV-2 for 14 days, and 68.6% were still positive. Most patients (84.3%) had an ECOG status of 4 and therefore were completely disabled and not able to carry out any self-care. During the time period of sPC involvement, oxygen supply was administered via a nasal cannula in 41.2% of the patients, a high flow oxygen supply in 35.3%, and 70.6% of the 51 patients received noninvasive ventilation at some point. Furthermore, 45.1% of the patients were mechanically ventilated, and 45.1% received ECMO; a few of them were also awake patients. The mean duration of the sPC treatment was 15.6 days with frequent contact with the multiprofessional sPC teams, e.g., physicians, nurses, psychologists, physiotherapists and other professions. Contact with spiritual care or creative care was performed upon individual request and need. In addition, the sPC team and psychologists cared for the relatives of patients. Information about the mean duration of each profession is available in Table 2. During sPC treatment, 62.8% of the patients died, two (3.9%) were referred to the sPC unit, and one third was discharged alive.

### 3.3. Symptom Control

#### 3.3.1. Symptom Burden

**Figure 1:**
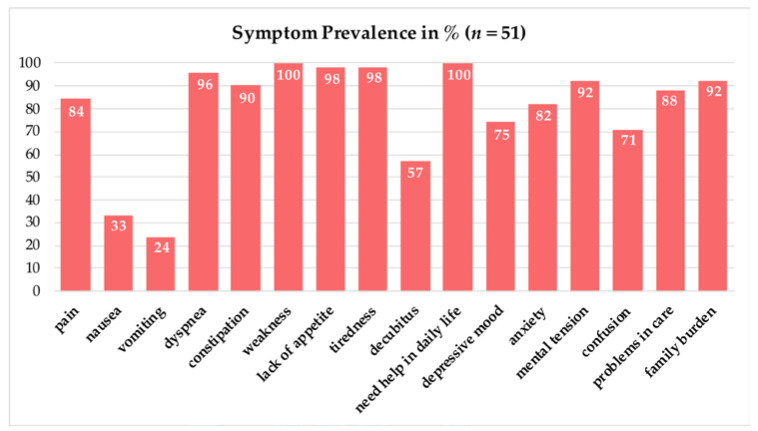
Symptom prevalence in percentage (%) assessed at the time of the first specialist palliative care contact.

When the sPC team first consulted the patients, all of the 51 patients suffered from weakness, followed by 98% (*n* = 50) of patients with tiredness and lack of appetite. The symptom prevalence of each of the 16 assessed symptoms is shown in Figure 1. Dyspnea was present in 96% (*n* = 49) of the patients and pain in 84% (*n* = 43). Family burden and mental tension were both present in 92% (*n* = 47) of the patients, and in 88% (*n* = 45), problems in the organization of care supply occurred. The lowest prevalence of the assessed symptoms were nausea (33%, *n*= 17) and vomiting (23%, *n* = 12). During the treatment period of sPC and the progression of the COVID-19 disease, eleven symptoms of the total sixteen were declining. The most notable symptom that was eased was mental tension with a prevalence reduction of 9.8%. In contrast, the prevalence of weakness, lack of appetite and tiredness did not change, while vomiting and decubitus became even more prevalent at the end of sPC care.

#### 3.3.2. Symptom Intensity

**Figure 2:**
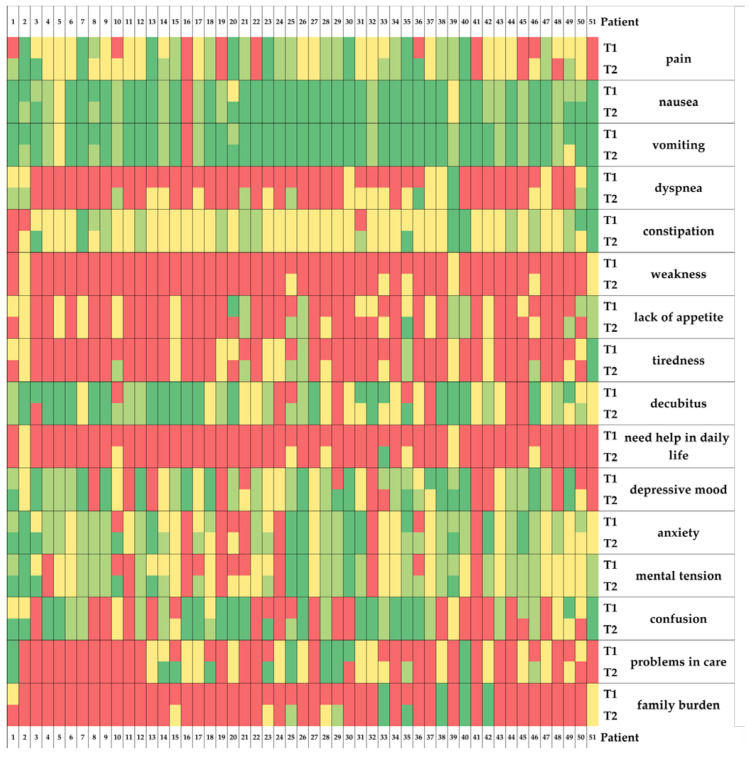
Heatmap indicating symptom intensity at the beginning (T1) and end (T2) of specialist palliative care in COVID-19 patients in the intensive care unit. Color-coding: no symptoms = green, light symptoms = bright green, moderate symptoms = yellow and severe symptoms = red.

**Figure 3:**
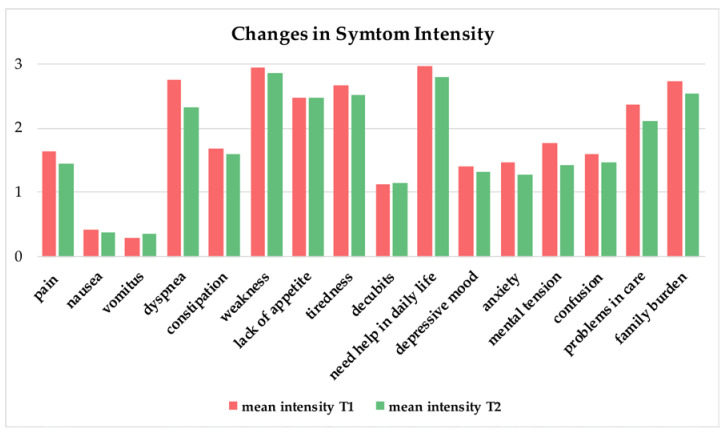
Changes in the mean values of each symptom intensity from the beginning (T1) to the end (T2) of specialist palliative care involvement (0 = no symptoms, 1 = light symptoms, 2 = moderate symptoms and 3 = severe symptoms).

The symptom intensity (no symptoms, light symptoms, moderate symptoms, severe symptoms) for each of the 16 assessed items and each patient are shown in a heatmap in Figure 2. The comparison of the summed-up symptom intensities (0 = no symptoms to 3 = severe symptoms) of the 16 assessed symptoms revealed a statistical significance between the beginning and end of sPC treatment (paired sample *t*-test; t(99) = 3.119; *p* = 0.003; d = 0.437). The biggest improvements were observed in dyspnea, mental tension and problems in the organization of care and anxiety. The change in mean values of each symptom intensity from the beginning (T1) to the end (T2) of sPC involvement is shown in Figure 3. A figure showing the differences in symptom intensities in the process of sPC involvement can be found in the supplementary material section (Figure S1: Differences in symptom intensities of the 16 symptoms throughout the sPC process).

## 4. Discussion

The present study investigates the characteristics and symptoms in COVID-19 ICU-patients treated with sPC from a palliative care perspective using the assessment tools ECOG and MIDOS. The main findings are: (1) prior to hospital admission, the patients were mostly living at home and had common comorbidities such as arterial hypertension and oncological diseases; (2) sPC involvement was frequent when patients were in a disabled condition or on ECMO therapy (43.4% sPC in ECMO patients) and had a high mortality rate (62.8%); (3) all members of the multiprofessional sPC team were involved; (4) the symptom burden with weakness and tiredness was high; however, a reduction in symptom burden was achieved, e.g., in mental tension; and (5) overall symptom intensity measured as the summed-up intensities of the 16 assessed symptoms decreased through sPC consultations and COVID-19 intensive care treatment.

Within the framework of the German program “Palliative care in Pandemics” (PallPan), the impact of the pandemic on general and sPC has been investigated [43]. General practitioners providing nonspecialist PC reported that the quality of the end-of-life care of their patients was maintained throughout the pandemic and reported their observed fear of loneliness among patients in nursing homes, private homes and hospitals [19]. An investigation of specialist outpatient PC reported changes in the care of patients due to restrictions in visiting hours and highlights the important role of outpatient sPC care in COVID-19 patients not being admitted to hospitals [21]. Inpatient sPC was also part of the PallPan project [23,43,44]. In both general and specialist inpatient PC, the increased hygiene standards with the mandatory personal protective equipment, together with impaired communication, restricted visiting hours and hampered interdisciplinary cooperation, were considered burdensome [23]. Overall, the PallPan project addressed multiple aspects of PC such as the infrastructure, patients with and without COVID-19, the relatives, mourning and the healthcare professionals. However, a focus on the ICU setting is lacking in this joint project. Sheehan and colleagues have investigated critically ill COVID-19 patients requiring ICU [34]. In their cohort, 39% received an inpatient sPC consultation, and these were more likely to be older and more affected by diseases. In our study, 19.5% of all ICU hospitalized COVID-19 patients received sPC, which is comparable to other investigations regarding all hospitalized patients [31,37]. In our cohort, 62.8% of the patients died during sPC involvement, which is similar to the mortality rate in the study by Sheehan and colleagues (56.3%, with 57.7% of them receiving sPC). In both studies, only the in-hospital mortality was assessed. They highlight the importance of sPC use in high-risk ICU COVID-19 patients and remark on its underutilization. The application of PC in the ICU setting as a complementary and simultaneous approach needs to be implemented [45].

At the beginning of the pandemic, the residents of nursing homes were considered especially vulnerable to COVID-19 [46]. However, our study supports the finding that COVID-19 affects people of all ages and states of health rather than only very old patients with pre-existing diseases living in nursing facilities. The patients included in our study were on average 69 years old with a range between 42 and 94 years and mostly living at home before hospital admission due to COVID-19. The common comorbidities of the COVID-19 patients were those that are common in the whole population: arterial hypertension, coronary heart disease, obesity and diabetes. One quarter of the patients suffered from cancer. Until first sPC contact when the patients were already severely ill, only 25% completed advance directives, and 45% designated a healthcare proxy. These data are in line with an investigation that questioned ICU patients about existing care planning documents (advance directive: 29.4%, designated healthcare proxy: 38.6%) [47]. Clinicians and patients should be aware of the importance of advance care planning. The patients of our cohort are comparable to another study investigating the patient characteristics of sPC COVID-19 patients [34]. Of the 23 patients on ECMO, 56.5% died during sPC treatment which is lower than the overall mortality rate in our cohort. A recently published meta-analysis reported an overall mortality of patients with COVID-19 receiving ECMO at 39% [48]. Our rate might be higher as it represents the very beginning of the COVID-19 pandemic, and the included patients receiving sPC might be more severely ill than ECMO patients without sPC [34]. In addition, the definition of a COVID-19-associated acute respiratory distress syndrome requiring ECMO therapy was a dynamic process throughout the pandemic. However, these authors support sPC involvement in all ECMO COVID-19 patients.

The special role of sPC in COVID-19 treatment is also due to the increasing treatment of fully awake patients with EMCO, which is demanding and represents new challenges [49]. This is challenging for the ICU staff, as they usually treat patients under sedation, and the sPC teams can particularly support here. Our data give an overview about the symptoms of COVID-19 patients in the ICU and can therefore prepare the sPC teams for their possible work with these patients. Although predictors of sPC need in late COVID-19 patients have already been investigated, more studies are needed to identify sPC need trigger criteria for an earlier and more comprehensive integration. As our study shows, patients had contact with the multidisciplinary members of the sPC team, and their families and friends were also supported. The presented symptoms and implementation highlight the special role of sPC in the COVID-19 ICU context. A qualitative study showed the massive burden for the relatives of ventilated COVID-19 patients [50]. In addition, clinicians can be relieved through sPC involvement, as, for example, moral distress is an urgent issue to be addressed [51].

Most therapeutic recommendations for COVID-19 therapy such as the WHO “Clinical management: living guidance” as well as PC-specific recommendations in the review by Beng and colleagues mainly focus on physical and mental symptom management [28,52]. However, our study used the MIDOS assessment at the beginning and end of sPC involvement. To our knowledge, this is the first study that evaluates the symptoms of ICU COVID-19 patients from a palliative care perspective with this assessment tool. Symptoms like weakness, tiredness, lack of appetite and mental tension were more prevalent than dyspnea. In addition, the assessment of the prevalence of family burden and problems in the organization of care are important to address. The symptom intensity significantly improved over the time of sPC involvement. Of course, next to sPC treatment, COVID-19 symptom intensities may have also decreased due to the specific COVID-19 and ICU treatment. Nevertheless, the biggest improvements observed, in addition to dyspnea, were in the reduction in mental tension and problems in the organization of care and anxiety. The symptom prevalence of weakness, lack of appetite and tiredness did not change in the study period; the reasons might be that the patients were still in the ICU at the end of sPC involvement, and addressing and improving these symptoms might need more time. Our study underlines the urgent need for sPC in COVID-19 patients to provide supportive care, both with physical and in nonphysical symptoms. However, as our data only give a very first insight in symptom change, further studies are needed to evaluate the statistical and clinical impact of sPC on the symptoms of COVID-19 patients.

### Limitations

There are several limitations to this study. This study is a retrospective study in a single institution and is therefore not generalizable. As we only included real-world data, a control group is lacking. In addition, symptom assessment was recorded for each patient at the beginning and the end of sPC involvement; further investigations with standardized survey dates would be favorable to provide reliable findings on the effect on symptoms. Furthermore, a change in symptom intensities without the influence of sPC was not investigated. In addition, the patients hospitalized in a university hospital ICU might not be comparable to other ICUs, where, for example, no ECMO treatment is possible. The specific number of awake ECMO patients was not determined in this study and therefore not analyzed any further. In our institution, no standardized and automatic sPC involvement was performed in COVID-19 patients, although ICU and sPC physicians are in agreement about the benefits. Still, sPC involvement only occurred on request by the intensivist. Further studies should evaluate the role of sPC on the long-term outcomes of COVID-19 patients and their families. In addition, the pandemic is dynamic with different phases of peaks and plateaus. Our collected data represent the time period from March 2020 until April 2021 with three waves in Germany. Since then, many factors such as the COVID-19 disease, vaccinations, therapies and others have changed and improved. Lastly, our findings may not be generalizable to other health systems and other healthcare institutions as there might be a significantly higher or lesser burden of COVID-19 cases.

## 5. Conclusions

After almost three years of the COVID-19 pandemic, investigations regarding the optimal care for these patients in ICU and especially regarding sPC needs is of great relevance. sPC involvement in COVID-19 is still novel because the disease is not a definite life-limiting condition, but nevertheless, it shows a high mortality in severely affected patients. In this study, we observed an overall reduction in the symptom intensities with a focus on dyspnea, mental tension, anxiety and the organization of care. In addition, the families of patients were supported. However, the integration of sPC in COVID-19 patients was underutilized (51 of the total 262 patients). With our results, we aim to draw attention to the sPC need of these patients and encourage intensivists to require sPC consultations. Finally, our results may support sPC teams in the future to prepare for their work with COVID-19 patients.

## Figures and Tables

**Table 1:** Baseline patient characteristics.

Characteristic	Mean (SD)	*n* (%)
Age, years	69.51 (15.84)	
Sex, male/female		32 (62.7)/19 (37.3)
Place of living		
Home		42 (82.4)
Nursing home		7 (13.7)
Missing data		2 (3.9)
Relatives available, yes/no		48 (94.1)/3 (5.9)
Comorbidities		
Arterial hypertension		36 (70.6)
Coronary artery disease		20 (39.2)
Obesity		16 (31.4)
Oncological disease		13 (25.5)
Diabetes mellitus		10 (19.6)
Dementia		6 (11.76)
Chronic lung disease		5 (9.8)
Care planning		
Advance directive		13 (25.5)
Health care proxy		23 (45.1)

SD, standard deviation.

**Table 2:** Performance status at first specialist palliative care (sPC) contact, COVID-19 treatment, sPC involvement and outcomes in COVID-19 patients in the intensive care unit receiving sPC.

Characteristic	Median (IQR)	*n* (%)
ECOG *		
2		1 (2)
3		7 (13.7)
4		43 (84.3)
COVID-19 treatment		
Noninvasive ventilation		36 (70.6)
Mechanical ventilation		23 (45.1)
ECMO *		23 (45.1)
Intravenous catecholamines		23 (45.1)
Dialysis		17 (33.3)
Opioids		29 (56.9)
Durations in days (d) or minutes (min)		
COVID-19 confirmation until sPC contact (d)	14 (11)	
sPC treatment in total (d)	13.5 (10.5)	
Contact with physicians (min)	140 (82.5)	
Contact with nurses (min)	120 (82.5)	
Contact with psychologists (min)	50 (82.5)	
Contact with physiotherapists (min)	110 (257.5)	
Contact with relatives to psychologists (min)	30 (50)	
Outcomes		
Deceased		32 (62.8)
Palliative care unit		2 (3.9)
Discharge		15 (29.4)
Missing data		2 (3.9)

* ECOG = Eastern Cooperative Oncology Group; ECMO = extracorporeal membrane oxygenation. SD, standard deviation.

## Data Availability

The dataset used and analyzed in this present study is available from the corresponding author on reasonable request.

## Supplementary Materials

The following supporting information can be downloaded at https://www.mdpi.com/article/10.3390/jcm11195925/s1: Figure S1: Differences in symptom intensities of the 16 symptoms throughout the sPC process.
