# Supplementary material for: Specialist Palliative Care Consultations in COVID-19 Patients in the ICU—A Retrospective Analysis of Patient Characteristics and Symptoms at a German University Hospital
Source: J Clin Med. 2022 Oct 7;11(19):5925. doi: 10.3390/jcm11195925 (PMC9571329; doi:10.3390/jcm11195925)
Supplement: Supplementary file 1 [file jcm-11-05925-s001.zip › Figure S1.pdf]

| Patient | pain | nausea | vomiting | dyspnea | constipation | weakness | lack of appetite | tiredness | problems in care | need help in daily life | depressive mood | anxiety | mental tension | confusion | problems in care | family burden |
|---|---|---|---|---|---|---|---|---|---|---|---|---|---|---|---|---|
| 1 | 0 | 0 | 0 | 0 | 0 | 0 | 0 | 0 | 0 | 0 | 0 | 0 | 0 | 0 | 0 | 0 |
| 2 | 0 | -1 | 0 | -1 | 2 | 0 | 2 | 1 | -1 | 0 | -1 | 1 | 0 | 1 | 1 | 0 |
| 3 | -1 | -1 | 2 | 0 | 0 | 0 | -2 | -1 | 2 | 0 | 0 | 0 | 0 | 2 | -1 | 0 |
| 4 | 1 | 0 | 0 | 0 | 0 | 0 | 0 | 0 | 0 | 0 | 0 | 0 | 0 | 0 | 0 | 0 |
| 5 | 0 | 0 | 0 | 0 | 0 | 0 | 0 | 0 | 0 | 0 | 0 | 0 | 0 | 0 | 0 | 0 |
| 6 | -1 | 0 | 0 | -1 | 0 | -1 | -1 | -2 | 0 | -1 | 0 | 0 | 0 | 0 | -2 | 0 |
| 7 | 0 | 0 | 0 | 0 | 0 | 0 | 1 | 0 | 0 | 0 | 0 | 0 | 0 | 2 | 0 | 0 |
| 8 | 0 | 0 | 0 | 0 | 0 | 0 | 0 | 0 | 0 | 0 | 0 | 0 | 0 | 0 | 0 | 0 |
| 9 | 0 | 0 | 0 | 0 | 0 | 0 | 0 | 0 | 0 | 0 | 0 | 0 | 0 | 0 | 0 | 0 |
| 10 | 0 | 0 | 0 | 0 | 0 | 0 | 0 | 0 | 0 | 0 | 0 | 0 | 0 | 0 | 0 | 0 |
| 11 | 0 | 0 | 0 | 0 | 0 | 0 | 0 | 0 | 0 | 0 | 0 | 0 | 0 | 0 | 0 | 0 |
| 12 | 0 | 0 | 0 | 0 | 0 | 0 | 0 | 0 | 0 | 0 | 0 | 0 | 0 | 0 | 0 | 0 |
| 13 | 0 | 0 | 0 | 0 | 0 | 0 | 0 | 0 | 0 | 0 | -1 | -1 | -1 | 0 | 0 | 0 |
| 14 | 0 | 0 | 0 | 0 | 0 | 0 | 0 | 0 | 0 | 0 | 0 | 0 | 0 | 0 | 0 | 0 |
| 15 | 0 | 0 | 0 | 0 | 0 | 0 | 0 | 0 | 0 | 0 | 2 | 0 | 0 | 0 | 0 | 0 |
| 16 | -3 | 0 | 0 | -3 | 0 | 0 | 0 | 0 | 0 | 0 | -1 | -2 | -2 | 0 | 0 | 0 |
| 17 | 0 | 0 | 0 | -1 | -2 | -1 | -2 | 0 | -3 | -1 | -1 | 1 | 0 | 0 | -2 | -3 |
| 18 | 0 | 0 | 0 | 0 | 0 | 0 | 0 | 0 | 0 | 0 | 0 | 0 | 0 | 0 | 0 | 0 |
| 19 | -1 | 0 | 0 | -1 | 0 | -1 | -1 | -1 | 2 | -3 | 2 | 0 | 0 | 0 | -1 | 0 |
| 20 | 0 | 0 | 0 | -1 | 0 | 0 | 1 | 0 | 0 | 0 | 0 | 0 | 0 | 0 | 0 | 0 |
| 21 | 0 | 0 | 0 | -1 | -2 | 0 | 1 | 0 | 2 | 0 | 0 | 0 | 0 | 0 | 0 | 0 |
| 22 | 0 | 0 | 0 | 0 | 0 | 0 | 0 | 0 | 0 | 0 | 0 | 0 | 0 | 0 | 3 | 0 |
| 23 | 0 | 0 | 0 | 0 | 0 | 0 | 0 | 0 | 0 | 0 | -3 | 0 | 0 | 0 | 0 | -2 |
| 24 | 0 | 0 | 0 | 0 | 0 | 0 | -1 | -1 | 0 | -1 | 0 | 0 | 0 | 0 | 0 | -1 |
| 25 | 0 | 0 | 0 | 0 | 0 | 0 | 0 | 0 | 0 | 0 | 0 | 0 | 0 | 0 | 0 | 0 |
| 26 | 0 | 0 | 0 | 0 | 0 | 0 | 0 | 0 | 0 | 0 | 0 | 0 | 0 | 0 | 0 | 0 |
| 27 | 0 | 0 | 0 | -2 | 0 | -1 | -2 | -2 | -2 | -1 | 0 | 0 | 0 | -2 | 0 | -2 |
| 28 | 0 | 0 | 0 | 0 | 0 | 0 | 0 | 0 | 0 | 0 | 0 | 0 | 0 | 0 | 0 | 0 |
| 29 | 0 | 0 | 0 | -1 | 0 | 0 | 0 | 0 | 0 | 0 | 0 | -1 | -1 | -3 | -3 | -1 |
| 30 | 0 | 0 | 0 | 0 | 0 | 0 | 0 | 0 | 0 | 0 | 0 | 0 | 0 | 0 | 0 | 0 |
| 31 | 0 | 0 | 0 | -2 | 0 | 0 | 0 | -2 | 0 | 0 | -1 | 0 | -1 | 0 | 0 | 0 |
| 32 | 0 | -2 | -1 | 0 | 0 | 0 | 3 | 1 | 0 | 0 | 0 | -1 | -1 | 0 | 0 | 0 |
| 33 | 0 | 0 | 0 | 0 | 0 | 0 | 0 | 0 | 0 | 0 | 0 | 0 | 0 | 0 | 0 | 0 |
| 34 | 0 | 0 | 0 | 0 | 0 | 0 | 0 | 0 | 0 | 0 | 0 | 1 | 0 | -1 | -3 | 0 |
| 35 | 0 | 0 | 0 | -1 | 0 | 0 | 0 | 0 | 0 | 0 | 0 | -1 | -2 | 0 | 0 | 0 |
| 36 | 0 | 0 | 0 | 0 | 0 | 0 | 0 | 0 | 0 | 0 | 0 | 0 | 0 | 0 | 0 | 0 |
| 37 | 0 | 0 | 0 | 0 | 0 | 0 | 0 | 0 | 0 | 0 | 0 | 0 | 0 | -1 | -3 | -1 |
| 38 | -1 | 0 | 0 | -1 | 0 | 0 | 0 | 0 | 0 | 0 | 0 | -1 | -1 | 0 | -2 | 0 |
| 39 | 0 | 0 | 0 | -1 | 0 | 0 | 0 | 0 | 0 | 0 | 0 | 0 | -2 | 0 | 0 | 0 |
| 40 | 0 | 0 | 0 | 0 | 0 | 0 | 0 | 0 | 0 | 0 | 0 | 0 | 0 | 0 | 0 | 0 |
| 41 | 0 | 0 | 0 | 0 | 0 | 0 | 0 | 0 | 0 | 0 | 0 | 0 | 0 | 0 | 0 | 0 |
| 42 | -1 | 0 | 0 | -2 | 0 | 0 | 0 | -2 | -2 | -1 | 0 | -3 | -3 | 0 | 0 | 0 |
| 43 | 0 | 0 | 0 | 0 | 0 | 0 | 0 | 0 | 0 | 0 | 0 | 0 | 0 | 0 | 0 | 0 |
| 44 | 1 | 1 | 1 | 0 | 1 | 0 | 0 | 0 | 0 | 0 | 0 | 0 | 0 | 0 | 0 | 0 |
| 45 | 0 | 0 | 0 | 0 | 0 | 0 | 0 | 0 | 0 | 0 | 0 | 0 | 0 | 0 | 0 | 0 |
| 46 | 0 | 0 | 0 | 0 | 0 | 0 | 0 | 0 | 0 | 0 | 0 | 0 | 0 | 0 | 0 | 0 |
| 47 | 0 | 0 | 0 | 0 | 0 | 0 | 0 | 0 | 0 | 0 | 0 | 0 | 0 | 0 | 0 | 0 |
| 48 | 0 | 0 | 0 | 0 | 0 | 0 | 0 | 0 | 0 | 0 | 0 | 0 | 0 | 0 | 0 | 0 |
| 49 | -2 | -1 | 0 | 0 | -2 | 0 | 0 | 0 | 3 | 0 | 0 | -2 | -2 | 0 | 0 | 0 |
| 50 | 0 | 1 | 1 | -1 | -1 | 0 | 0 | 0 | 0 | 0 | 0 | 0 | 0 | -2 | 0 | 0 |
| 51 | -2 | 0 | 0 | -1 | 0 | 0 | 1 | 1 | 0 | 0 | -1 | -1 | -1 | -2 | 0 | 1 |
| total | -10 | -3 | 3 | -21 | -4 | -4 | 0 | -8 | 1 | -8 | -5 | -10 | -17 | -6 | -13 | -9 |

**Figure S1. Differences in symptom intensities of the 16 symptoms throughout the sPC process.** This figure displays in total and for each patient (n = 51) the change (-3 = most reduced to 3 = most increased) in symptom intensities (0 = no symptoms, 1 = light symptoms, 2 = moderate symptoms, 3 = severe symptoms) of the 16 assessed symptoms in the process of sPC involvement (from first contact to the end of sPC treatment).
